# The Midlife Women’s Health Study – a study protocol of a longitudinal prospective study on predictors of menopausal hot flashes

**DOI:** 10.1186/s40695-017-0024-8

**Published:** 2017-08-17

**Authors:** Ayelet Ziv-Gal, Rebecca L. Smith, Lisa Gallicchio, Susan R. Miller, Howard A. Zacur, Jodi A. Flaws

**Affiliations:** 1 School of Health Sciences, Massey University, Palmerston North, New Zealand; 2 Department of Pathobiology, University of Illinois, Urbana, Illinois USA; 3 Epidemiology and Genomics Research Program, Division of Cancer Control and Population Sciences, National Cancer Institute, Bethesda, Maryland USA; 4 Johns Hopkins University School of Medicine, Baltimore, Maryland USA; 5 Department of Comparative Biosciences, University of Illinois, 2001 S. Lincoln Avenue, Urbana, Illinois 61802 USA

**Keywords:** Hot flash, Menopausal transition, BMI, Cigarette smoking, Race, Study protocol

## Abstract

**Background:**

The Midlife Women’s Health Study (MWHS) was developed to address some of the gaps in knowledge regarding risk factors for hot flashes among generally healthy midlife women during their menopausal transition. This manuscript describes the methods from the study and the main findings that were published to date, with a focus on predictors of hot flashes. This study was initially funded to test the hypothesis that obesity is associated with an increased risk of hot flashes through mechanisms that involve ovarian failure, altered sex steroid hormone levels, and selected genetic polymorphisms.

**Methods/Design:**

The MWHS was conducted between 2006 and 2015 as a prospective longitudinal population-based study of generally healthy midlife women (ages 45 to 54 years) during their natural menopausal transition. Women were eligible if they had intact uteri and both ovaries and reported having at least 3 menstrual periods in the last 12 months. Exclusion criteria included pregnancy, cancer, and use of hormonal/hormone-like supplements. Overall, 780 women were recruited into the study. The majority of study participants were followed for 4 to 7 years. At annual visits, women donated blood and urine samples, completed questionnaires, had a vaginal ultrasound, and had their anthropometric measurements taken.

**Discussion:**

Several risk factors for menopausal hot flashes were identified or confirmed, including older age, perimenopausal status, current and former cigarette smoking, lower estradiol levels, lower progesterone levels, black race, and depressive symptoms. Factors that were associated with decreased odds of hot flashes included moderate alcohol consumption and more than 5 years of cessation of cigarette smoking. Body mass index was not associated with hot flashes. The MWHS has provided important information regarding hot flashes. The study methods are rigorous and can be easily adopted by research groups investigating naturally occurring menopausal hot flashes.

## Background

Hot flashes are the most common symptom reported by women during their menopausal transition [1, 2]. They are described as sudden transient periods of intense heat in the upper parts of the body, arms, and face. Hot flashes are often followed by flushing of the skin, profuse sweating, chills, palpitations, and anxiety [1]. In the United States, it is estimated that the health care expenditures due to hot flashes can be as high as 339 million dollars per year [3]. Additionally, symptomatic women can suffer from difficulties in overall daily functioning [4]. Despite the high prevalence of menopausal hot flashes and the overall public burden, the etiology and longitudinal changes/dynamics of natural occurring and untreated hot flashes are still unknown.

Few risk factors have been consistently reported to be associated with the occurrence of midlife hot flashes [5, 6]. Cigarette smoking, later menopausal stage, and low estrogen levels have been associated with an increased risk of hot flashes [5, 6]. Additionally, moderate alcohol consumption has been found to be associated with a reduced risk of hot flashes, whereas body mass index (BMI) and body fat composition have been shown to be associated with both increased and decreased risk of hot flashes [5, 6]. However, previous studies were mostly cross-sectional, investigated hot flashes with a limited number of questions, encompassed limited time periods, or focused on treatment efficacy rather than on untreated hot flashes. A recent study by Avis et al. examined hot flashes dynamics, but it focused only on frequent hot flashes instead of examining other aspects of hot flashes (e.g., severity, less frequent hot flashes) [7]. Thus, overall, there was a need for a study such as the Midlife Women’s Health Study (MWHS) to examine the dynamics of natural occurring and untreated hot flashes over time. This information was needed to fill some of the gaps in our knowledge about hot flashes, including events that predispose women to develop hot flashes, the estimated duration of hot flashes, and changes in hot flashes severity over time.

**Fig. 1:**
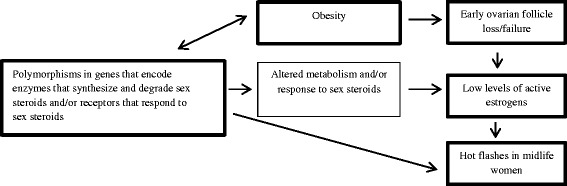
The MWHS study was designed to test the hypothesis that obesity is associated with hot flashes through: a) early ovarian follicle loss/failure, b) selected genetic polymorphisms in the genes that encode enzymes that synthesize and degrade sex steroids and/or the receptors that allow tissues to respond to sex steroids, or c) mechanisms involving early follicle loss/failure, altered sex steroid hormone levels, and genetic polymorphisms in genes that encode enzymes that synthesize and degrade sex steroids and/or receptors that respond to sex steroids (this part of the study is yet to be conducted)

The MWHS was conducted between 2006 and 2015 as a prospective longitudinal population-based study of generally healthy midlife women recruited during their menopausal transition. The overall goal of the study was to expand findings from a previous cross-sectional study that was conducted by the same team [8–23], while focusing on the mechanisms by which obesity is associated with an increased risk of hot flashes. Specifically, the MWHS was developed to test the hypothesis that obesity is associated with an increased risk of hot flashes through mechanisms that involve ovarian failure, altered sex steroid hormone levels, and selected genetic polymorphisms. The study working model is shown in Fig. 1.

Specific analyses that have been completed to date in the MWHS include: 1) identification of risk factors associated with longer duration of hot flashes and the time of peak hot flashes severity [24]; 2) examination of the associations between demographic characteristics, health behaviors, hormone concentrations, and the experience of any, current, more severe, and more frequent midlife hot flashes [25]; 3) examination of the association between quitting smoking and midlife hot flashes [26]; 4) examination of the associations between BMI, BMI change, and weight change and midlife hot flashes [27]; 5) examination of whether higher urinary levels of phthalate metabolites are associated with an increased risk of midlife hot flashes [28]; 6) examination of the associations between BMI, cigarette smoking, alcohol intake, and hormone concentrations with ovarian volume among midlife women [29]; and 7) identification of factors associated with sexual activity during the menopausal transition [30].

In the next sections we describe in detail the main methods that were used during the MWHS for recruitment of the study participants, handling of biological samples, data collection, and analyses. Additionally, we describe the main findings that have been published to date along with a concise discussion of the results.

## Methods

### Study design

The Midlife Women’s Health Study (MWHS) was a prospective longitudinal population-based study. It was innovative as it included generally healthy midlife women who were either late premenopausal or perimenopausal women. The inclusion criteria allowed the research team to examine, prospectively, the associations between specific variables (e.g., demographic, health habit, and clinical factors) and the occurrence, frequency, severity, and duration of hot flashes over time. Additionally, the detailed questionnaires completed by the study participants allowed for the examination of other commonly reported symptoms/issues during the menopausal transition, including sexual function, mood, and medical conditions (e.g., hypertension, allergies, diabetes).

### Sample selection and recruitment

The MWHS team used predetermined eligibility and exclusion criteria to ensure that during the initial recruitment period, women were not postmenopausal and were undergoing a natural process of reproductive aging. Specifically, women in the age range of 45 to 54 years were included as these women are typically perimenopausal and this is when women are most likely to have hot flashes [2]. Eligibility criteria also included having intact uteri and both ovaries; hence, only those women who were naturally undergoing the menopausal transition were eligible, whereas women who had surgical menopause were excluded from participation. Lastly, women who reported having at least 3 menstrual periods in the last 12 months were included, whereas women who did not have a menstrual period for ≥12 months were excluded because they are clinically considered postmenopausal [31].

**Fig. 2:**
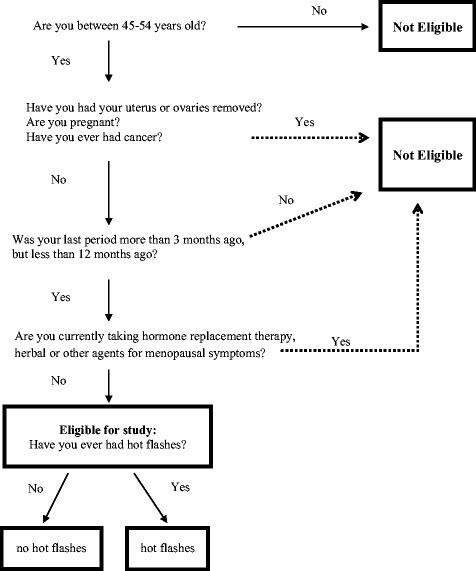
Potential participants in the MWHS were screened for eligibility based on the described selection algorithm. Specifically, women between the ages of 45 and 54 years, with an intact uteri and both ovaries, not pregnant, who have not had cancer, who have had their last menstrual period within the last 12 month period, but not within the last 3 month period, and who have not used hormone replacement therapy, herbal or plant substances for treatment of hot flashes were eligible for the MWHS

Additional exclusion criteria were used to avoid known factors that may interfere with the natural menopausal transition and the natural occurrence of hot flashes. Specifically, currently pregnant women were excluded because the study focused on women who were transitioning from a reproductive to non-reproductive stage of their lives. Additionally, women currently using hormone therapies or oral contraceptives were excluded because the study focused on women’s natural experiences of hot flashes, and hormone therapies and oral contraceptives are often used to prevent/reduce hot flashes. Lastly, women with any history of cancer were excluded because chemotherapeutic agents used to treat cancer can deplete ovarian follicles and increase risk of hot flashes [32]. Figure 2 summarizes the eligibility strategy for initial recruitment of participants.

Mailing addresses of women aged 45 to 54 years residing in the Baltimore, Maryland metropolitan area (USA) and its surrounding counties were purchased from AccuData America (Fort Myers, FL, USA). Recruitment letters were then sent to addresses located nearest to the clinical site at Johns Hopkins, Greenspring Station and then in concentric circles out from the site until reaching the target number of enrollees. To avoid potential reporting bias, the study was presented as a general “Midlife Women’s Health Study.” Women who were interested in enrolling in the study were asked to call the clinic to obtain more information.

Once a woman called the clinic, a clinic staff member determined if the woman met the eligibility criteria. Based on a woman’s interest and eligibility, a baseline clinic visit was scheduled. At this baseline clinic visit, the woman was informed again of the general purpose of the study, and her questions were answered. All participants provided written informed consent according to procedures approved by the University of Illinois and Johns Hopkins University Institutional Review Boards and each woman received a copy of the consent form.

During the baseline clinic visit, each participant was asked to complete a detailed baseline study questionnaire, donate urine and blood samples for hormone measurements, and have her weight, height, waist and hip circumferences and blood pressure measured. In addition, each participant underwent a transvaginal 2D ultrasound to measure ovarian volume and follicle numbers. Generally, baseline clinic visits were scheduled in the mornings (8:30–10:00 AM) to minimize daily fluctuations in hormone levels between the participants. Women were also instructed to fast overnight to avoid any potential dietary effects on hormone levels.

Each participant was then asked to visit the clinic once a week on each of the 3 weeks following the baseline visit to provide additional blood and urine samples. At the fourth clinic visit (the last of the three weekly visits following the baseline visit), each woman also completed another shorter questionnaire. Further details about the questionnaires are provided below.

During the clinic visits, a staff member reviewed each questionnaire for completeness and recorded any medications that the participant was taking on a regular basis. Hot flash status was assigned using the participant’s answer to the question “Have you ever had hot flashes?” (“yes” = ever experienced hot flashes, “no” = never experienced hot flashes). After each visit, the participant was given $10 US to cover the expense of time and travel to the clinic, and was provided with a voucher for a snack after the fasting blood work.

These four consecutive weekly clinic visits were then repeated on a yearly basis throughout the woman’s participation in the study, with visits proceeding similarly to the first year as described above. During these visits, the clinic staff examined any change that potentially affected a woman’s eligibility for further participation in the study. Specifically, the study team discontinued follow-up of women who reported the current use of hormone therapy, ever had an oophorectomy and/or hysterectomy, or ever were diagnosed with cancer. Generally, women were followed over a total of 4 years because they became postmenopausal at the end of the 4 year follow up. However, some participants were followed for more than 4 years because they were not postmenopausal at the end of the 4th year follow up.

**Fig. 3:**
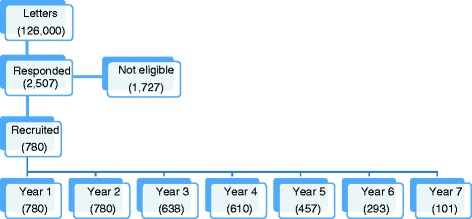
A flow chart describing the number of participants during the initial recruitment and the number of active participants per year

A total of 126,000 recruitment letters were mailed, 2507 women called the clinic for more information and were screened for eligibility. Of these women, a total of 780 women were recruited and were active participants during the first year of the MWHS. About 5.5% of 780 women withdrew after year 1 and approximately 3% dropped out after each subsequent year. Some of the reasons for withdrawal included lack of time, a medical issue, or the participant moving out of town. The study team discontinued follow-up of women who reported the use of hormone therapy (*n* = 30), had an oophorectomy and/or hysterectomy (*n* = 25), or were diagnosed with cancer (*n* = 12) [29]. Figure 3 provides a flow chart of women enrolled in the study.

If a woman missed a single visit or a year of visits, she was still asked to remain in the study and data from those skipped visits were considered missing. Lastly, to protect the participants’ privacy, each participant received a unique identification code. All records and data were stored in a locked file cabinet in a designated office and only personnel directly involved in the study had access to the files.

### Questionnaires

During the first visit and the last visit of each year of participation, women were asked to complete a self-administered questionnaire while seated in a private comfortable room. During the first visit, participants completed a 20-page, single-sided survey that took about an hour to complete. This detailed questionnaire contained questions regarding demographic information, reproductive history and menstrual cycle characteristics, hormonal and other supplement consumption, menopausal symptoms, medical and family history, and health behaviors such as smoking and alcohol use.

During the last yearly visit, the participant completed a condensed version of the questionnaire (9 pages, single-sided) that took about 30 min to complete. This survey assessed only factors that may have changed during the course of a 2 to 4-week time period (the time between the ‘same year’ visits). For example, the survey included questions on medical history, hot flashes history, smoking history, whereas factors such as birth date and race were excluded from the condensed version of the questionnaire.

### Anthropometric measurements

On the day of each visit, women were weighed without shoes in street clothing to the nearest 0.05 kg, rounding down, on a calibrated scale. Height was measured without shoes to the nearest 0.5 cm, rounding down, with a standard stadiometer. Body mass index (BMI) was calculated using the National Institutes of Health on-line BMI calculator [33]. Normal, overweight, or obese status was categorized as BMI less than 25 kg/m^2^, 25–29 kg/m^2^, and 30 kg/m^2^ or greater, respectively. Waist circumference was measured at the narrowest part of the waist. Hip circumference was measured at the fullest part of the hips.

### Measurement of ovarian volume and antral follicle numbers

Transvaginal ultrasounds were performed on each study participant on a yearly basis by a licensed, highly trained physician in the Department of Gynecology and Obstetrics at Johns Hopkins University. All transvaginal ultrasounds were performed using the 7.5 MHz transvaginal probe on a GE Logig 200 Alpha/Pro Model. All measurements were conducted without knowledge of the woman’s age, menopausal status, or hot flash status. Examination of the ovary was established by scanning from the outer to the inner margin. All follicles 2–10 mm in size were measured and counted in each ovary. Follicle size was calculated from 2 to 3 perpendicular measurements. The volumes of each follicle and ovary were calculated by applying formulas of an ellipsoid (LxWxDxpi/6). Total ovarian volume was obtained by summing the volumes of both ovaries.

### Blood collection and measurement of hormone levels

Blood was collected via venipuncture conducted by a trained phlebotomist. Aliquots of whole blood samples were either stored in −20 °C for future genetic analyses or were further processed for serum extraction. For serum extraction, samples were centrifuged at 2000 g for 20 min in a cooled centrifuge. After centrifugation, the serum was aspirated and stored at −70 °C until hormone analysis.

Because the participants were going through the menopausal transition, they had irregular cycles during the study. To minimize variability between measurements, samples were collected from fasting women, at the same time of day, but not on the same day of the cycle. Additionally, four different blood samples (one per week within a month) per each year of the study were collected. The values from these samples were averaged per year in the statistical analyses.

Serum concentrations of estradiol, testosterone, progesterone, and sex hormone-binding globulin (SHBG) were measured by enzyme-linked immunosorbent assays (ELISAs; DRG, NJ USA). All assays were performed without knowledge of participant characteristics by the same laboratory (Dr. Flaws, University of Illinois, Urbana, IL, USA). Each sample was quantified in duplicate within the same assay. Some samples were run in multiple assays to ensure that the assay values did not dramatically shift over time. Overall, the averaged inter-assay variability was less than 5%. A mean value, per participant, was used in all statistical analyses. When a participant’s hormone levels were below the limit of detection, a value between zero and the detectable limit (based on uniform distribution) was randomly assigned to ensure a more accurate estimate of the variance.

### Determination of menopausal status

Menopausal status was determined based on woman’s answers to several questions on the study questionnaire on menstrual cycle history (e.g., age at menarche, regularity of menstrual cycles, and number of menstrual cycles in the past year). Specifically, premenopausal women were those who experienced their last menstrual period within the past 3 months and reported 11 or more periods within the past year. Perimenopausal women were either those who experienced their last menstrual period within the past year, but not within the past 3 months or had their last menstrual period within the past 3 months and overall 10 or fewer periods within the past year. Postmenopausal women were those who had no menstrual periods within the past year. During the study, the follow-up of women who became postmenopausal (*n* = 120) was discontinued.

### Hot flashes variables

A detailed hot flash history was obtained through a series of questions in the study questionnaires. These specific questions have been used to collect data on hot flashes in the previous studies conducted by the MWHS team for more than 12 years [10, 11, 13, 15, 22, 27, 29, 34]. Women were asked: if they ever had hot flashes; whether they had experienced hot flashes within the last 30 days; the number of hot flashes experienced within the last 30 days; the age when hot flashes first occurred; the severity and frequency of the hot flashes; and the length of time a woman had been experiencing hot flashes.

Hot flashes severity was classified as moderate or severe if a woman had hot flashes that were described as a sensation of heat accompanied by sweating that may interrupt usual activity. A woman was classified as having mild hot flashes if she had hot flashes that were described as a warm sensation without sweating or disruption of usual activity. Hot flashes frequency was determined based on detailed questions on the occurrence of hot flashes. Specifically, the participants were asked if they experienced hot flashes every hour, every 2–5 h, every 6–11 h, every 12–23 h, 1–2 days per week, 5–6 days per week, 2–3 days per month, 1 day per month, less than 1 day per month, or never.

Time to peak severity was calculated as the difference between the age at which hot flashes were most severe and the age at which hot flashes were first experienced. By default, peak severity was the age at which hot flashes were reported to be most severe during the baseline visit. If, during the study, a woman reported a higher severity on a survey than reported on the baseline visit survey, the time of the later survey was considered to be the participant’s peak severity.

Additionally, women were asked whether any other female relatives (e.g., mother, sister, aunt) experienced hot flashes. Lastly, several questions inquired about quality of sleep and hot flashes experienced during the night (i.e., night sweats). Specifically, women were asked about the occurrence, number of events during the night (frequency), and severity (need to change clothes/sheets at night and frequency in a typical week) of night sweats.

### Sexual activity

Sexual activity was determined by several questions that inquired whether the participant was sexually active, her level of satisfaction in the case of being sexually active, and the reasons for not being sexually active (partner related or individual reasons). A subset of outcomes was generated based on items included in the Short Personal Experiences Questionnaire [35] such as frequency of sex, enjoyment of sex, arousal during sex, orgasm during sex, passion for partner, satisfaction with partner, pain during sex, lubrication during sex, and sexual fantasies. A variable for a group of the outcomes was created, and participant scores were calculated using Likert scale values (“1” = Not at all; “5” = A great deal).

### Lifestyle habits

Previous studies, including our preliminary cross-sectional study, indicated that cigarette smoking is associated with increased risk of hot flashes [11, 17, 36]. In the MWHS, cigarette smoking status was assessed using the questions: “Have you ever smoked cigarettes?” and “Do you still smoke cigarettes?” Cigarette smoking status was then categorized as current, former, and never. For smokers, information was also collected on the frequency, amount, and type of smoking.

Similarly, data on alcohol consumption were collected using the following questions: “During your entire life, have you had at least 12 drinks of any kind of alcoholic beverage?” and “In the last 12 months, have you had at least 12 drinks of any kind of alcoholic beverage?” Further queries for those responding affirmatively on having at least 12 drinks in the last 12 months were made to assess the average number of days per month that the woman drank and the number of drinks on those days.

Physical activity was assessed by the participant’s response to questions regarding their levels of activity at work and at leisure time. These included questions such as “At work, I sit/stand/walk/lift heavy loads/tired/sweat” [choices: never, seldom, sometimes, often, and always], “In comparison with others my own age, I think my work is physically” [choices: much heavier, heavier, as heavy, lighter, and much lighter], “In comparison with others my own age, I think my physical activity leisure time is” [choices: much more, more, as much, less, and much less], and “During leisure time, I play sport/watch television/walk/cycle” [choices: always, often, sometimes, seldom, and never].

### Mood/Emotional status

The experience of depressive symptoms was assessed using the Centers for Epidemiologic Studies – Depression Scale (CES-D) [37]. The study questionnaire included multiple questions in which women were asked to describe themselves during the visit at the clinic and during the past week. These questions included statements that best described what a woman felt (e.g., ‘I was happy’, ‘I thought my life had been a failure’, ‘I had crying spells’, ‘I had trouble keeping my mind on what I was doing’). The participant was asked to check the best fit on a given scale [choices: rarely, some of the time, moderately, most of the time].

### Other health related outcomes

The women were asked about their medical history using a series of several questions. The first question provided a list of selected illnesses/medical conditions of which the participant was asked to mark “Yes/No” and to indicate the age first diagnosed. The list included illnesses/conditions such as uterine fibroids, diabetes, epilepsy, and asthma. Other questions presented other symptoms that may be experienced by women in this age group such as incontinence, vaginal discharge, and headaches. The participant was asked to mark how frequently she had experienced each symptom during the past year [choices: never, rarely, sometimes, frequently, and regularly]. Lastly, a staff member queried the participants on medication use and that information was documented separately.

The questionnaire also included questions on hormone replacement therapy and herbal supplements. This allowed confirmation that participants were not taking hormonal or herbal agents. The questionnaire also included questions on the participant’s reproductive history (e.g., number of pregnancies, use of oral contraceptives).

### General information

Employment status was categorized as employed (either full-time or part-time) and not employed. Data on occupation and total family annual income, marital status, race/ethnic background, and education level were also collected on the detailed questionnaire administered at the first visit each year.

### Phthalate metabolite levels

Phthalates can be readily found in personal care products and are considered endocrine disrupting chemicals [38–40]. Because women are likely to use these phthalate-containing products, a subset of samples from 195 participants (96 with hot flashes and 99 without hot flashes) was evaluated for urine phthalate metabolites levels. To minimize potential confounding, this subset of samples included only nonsmokers and white women with similar BMIs. Urine samples were analyzed by isotope dilution high-performance liquid chromatography negative-ion electrospray ionization-tandem mass spectrometry (HPLC-MS/MS) at the Environmental Health Laboratory & Trace Organics Analysis Center, School of Public Health at the University of Washington. Detailed methods are described by Ziv-Gal et al. [28].

### Statistical analyses

In MHWS publications, data have been analyzed using various statistical approaches. The approaches included, but are not limited to, univariate and bivariate analyses [24, 25], logistic regression and generalized estimated equation models [24–29], survival analysis [24], and Bayesian network analysis [24, 30]. For each analysis, potential effect modifiers and confounders were examined as described in detail in the published manuscripts.

## Published results to date

### Sample characteristics and hot flashes

At baseline, 45.4% of the study participants reported experiencing hot flashes [27]. The majority of these women reported experiencing hot flashes in the previous 30 days (72.3%) [27]. Approximately 55.7% of women reported hot flashes that were moderate in severity, and about a quarter of those women with hot flashes reported experiencing them daily (23.2%) or weekly (26.2%) [27]. The majority of women had experienced hot flashes for more than 1 year (63%) [25]. In the longitudinal analysis, women experienced hot flashes over 2.5 years on average when including only those who reported an end to their hot flashes within the first 4 years of the study [24]. When including all women during all years of study, hot flashes duration was 6 years on average [24].

At baseline, older age, higher education level, having depressive symptoms, and use of anti-hypertensive medications were significantly associated with increased odds of hot flashes [27]. In contrast, marital status and physical activity were not significantly associated with hot flashes outcomes [27]. In the longitudinal analysis, shorter mean duration of hot flashes (i.e., the difference between the age first experiencing hot flashes and the age first reported not having hot flashes) was significantly associated with higher education level during the study period [24]. Additionally, longer mean duration was associated with delayed time to peak severity [24].

### Menopausal status, hormone levels, and hot flashes

At baseline, women experiencing hot flashes were more likely to be of perimenopausal status (56%) [27] and to have significantly lower estradiol and progesterone levels compared to women without hot flashes [25]. Additionally, mean ovarian volume was significantly lower in women experiencing hot flashes compared to women without hot flashes [25]. Testosterone levels were similar between women with and without hot flashes [25]. In the longitudinal analysis, shorter mean duration (in years) of hot flashes was significantly associated with higher estradiol and progesterone levels among women with hot flashes during the study period [24]. Higher progesterone levels were associated with decreased time to peak severity [24]. Lastly, similar to baseline results, testosterone levels were not statistically associated with hot flashes [24].

### Weight, BMI, and hot flashes

The main objective of the funded MWHS study was to evaluate the association between body weight and menopausal hot flashes. Baseline data of MWHS indicated that BMI was not associated with any of the hot flashes outcomes [27]. Similarly, longitudinal analysis indicated no association between BMI, BMI change, or weight change and any of the hot flashes outcomes [27].

### Race and hot flashes

At baseline, race was not associated with menopausal hot flashes [24, 25, 27]. However, race was significantly associated with experiencing hot flashes over time [24]. Specifically, black women were more likely to have hot flashes over a longer duration (in years) when compared to white women; however, white women had a significantly earlier peak of hot flashes severity compared to black women [24].

### Cigarette smoking and hot flashes

At baseline, both current and former cigarette smoking were significantly associated with increased odds of hot flashes outcomes, independent of estradiol levels [27]. Similarly, in the longitudinal analysis, cigarette smoking was significantly associated with a longer mean duration (in years) of hot flashes during the study period [24].

The association between quitting cigarette smoking over time and hot flashes was further examined in the MWHS [26]. Findings from the MWHS were suggestive for a differential effect of cigarette smoking on hot flashes outcomes. Specifically, women who quit smoking for more than 5 years were less likely to suffer from hot flashes (any, severe, or frequent) compared to women who continued smoking; however, they remained at higher risk for having any, severe, and frequent hot flashes compared to women who never smoked cigarettes [26].

### Alcohol consumption and hot flashes

At baseline, higher alcohol consumption was associated with decreased odds of menopausal hot flashes [25]. In the longitudinal analysis, women who consumed at least 12 drinks in the previous year had significantly shorter hot flash duration and shorter mean time to peak severity (in years) compared to women who consumed less than 12 drinks in the previous year [24].

### Sexual activity and hot flashes

When comparing the frequency of hot flashes and sexual activity, women with less frequent hot flashes (weekly) were more likely to be sexually active than those with more frequent hot flashes (daily). These results were independent from having a partner [30].

### Phthalate metabolite levels and hot flashes

The association between levels of urinary phthalates and the risk of menopausal hot flashes was statistically analyzed using baseline data from a subset of participants as described above [28]. The results indicated that levels of phthalate metabolites commonly found in personal care products were positively associated with an increased risk of ever experiencing hot flashes, hot flashes in the past 30 days, and more frequent hot flashes [28].

### Findings independent of hot flashes

Given the detailed nature of the MWHS questionnaire, investigators were able to examine a variety of outcomes other than hot flashes. The key findings from some of these analyses are described below.

#### Ovarian volume

These analyses were based on data collected at baseline and the fourth year of the MWHS. Results indicated that a significant reduction in ovarian volume was associated with older age and later stage of the menopausal transition [29]. Additionally, ovarian volume was found to be significantly and positively associated with estradiol levels in the entire cohort and when stratified by race [29]. In contrast, progesterone levels were significantly and positively associated with ovarian volume only among white women [29]. Lastly, BMI, alcohol intake, and cigarette smoking were not associated with ovarian volume at baseline or at the fourth year of the study. These results were observed for the entire cohort and when stratified by baseline menopausal status [29].

#### Age at menarche and midlife obesity

An analysis of baseline MWHS data showed that age at menarche was significantly associated with midlife obesity, independent of testosterone and estradiol concentrations in adulthood [41]. Other variables significantly associated with higher BMI and obesity were black race, perimenopausal status, lower education level, higher weight at the age of 18, and never smoking [41].

#### Sexual activity

Several factors were found to be positively associated with sexual activity during the menopausal transition. Such factors included higher estradiol levels, higher income, heavy physical work, and better mental condition (i.e., less depressed, less fatigue, and less irritable) [30]. In contrast, some variables such as alcohol consumption, race, testosterone levels, and the amount of smoking cigarettes were not significantly associated with any of the sexual activity outcomes [30].

## Discussion

The MWHS followed midlife women over time to examine their experience of hot flashes in a detailed manner. The study design allowed for the longitudinal examination of various factors (e.g., race, BMI, hormone levels) that were shown to be associated with hot flashes in cross-sectional studies. It also allowed for the examination of factors that could not be assessed in cross-sectional studies (e.g., hot flashes peak severity, quitting cigarette smoking). Overall, the results from the MWHS, as well as others, strongly suggest that menopausal symptoms are likely multi-factorial [42, 43].

Some of the longitudinal results of the MWHS are consistent with those reported in other longitudinal studies. Specifically, the MWHS study showed that menopausal hot flashes were significantly associated with age, menopausal stage, education level, race, some hormonal changes, and cigarette smoking [24]. Similarly, the Study of Women’s health Across the Nation (SWAN) showed that education level, age, race, smoking cigarettes, and hormone levels were associated with menopausal hot flashes [6, 45]. The Penn Ovarian Aging Study showed that race, menopausal stage, hormone levels, education level, and smoking cigarettes were associated with menopausal hot flashes [44, 46]. The Australian Longitudinal Study on Women’s Health showed that education levels and menopausal stage were associated with menopausal hot flashes [47]. Lastly, the Norwegian Hordaland Women’s Cohort Study showed that education levels and smoking status were associated with hot flashes [48]. A unique contribution of the MWHS is the observation that some personal interventions such as quitting smoking may be beneficial in reducing the odds of hot flashes [26].

In contrast, some results from the MWHS are not in agreement with findings from other longitudinal studies and hence necessitate further investigation. For example, findings from the MWHS indicated that neither BMI change nor change in weight during the menopausal transition were associated with the risk of hot flashes [27], whereas findings from SWAN indicated that body fat gain measured by bioelectrical bio-impedance was associated with increased odds of hot flashes [49]. Differences in study design (e.g., cohorts, evaluation, collection methods) can explain some of these differences in results. For example, the cohort compositions were different with respect to their geographical location and ethnic representation. The MWHS recruited mostly white and black women from the Baltimore area [25, 45, 50], whereas SWAN [45] recruited participants from multiple locations around the United States and included a greater representation of Hispanic women compared to the MWHS. In addition, various cohort studies differ in the duration of the study. For example, the Penn Ovarian Aging Study was conducted over 16 years (*n* = 255), whereas the MWHS was conducted over 7 years (*n* = 780). Further, some of the outcomes under study were calculated differently. In the MWHS, timing of hot flashes peak severity was calculated relative to the time that hot flashes were first experienced [24], whereas in the Penn Ovarian Aging Study, timing of peak severity was calculated relative to the timing of the final menstrual period [44].

The data from the MWHS showing that BMI was not associated with hot flashes outcomes were surprising because our previous cross-sectional study, which formed the basis for the development of the MWHS, showed that obesity was associated with increased odds of hot flashes [9, 15, 27]. The reasons for the discrepant findings between the MWHS and our previous cross-sectional study are unclear. It is possible that the perimenopausal women in the MWHS were later in perimenopause than women in our previous cross-sectional study. Although the MWHS obtained information on the participants’ menopausal stage, it was not possible to know from our data how early the participants were in each stage and how this compares to other studies. Thus, we can only speculate that reported differences in the association between BMI and hot flashes among studies may be due to differences in where participants were in each stage of the menopausal transition or due to other unknown factors that were not evaluated.

The MWHS had several limitations. The results of the MWHS are generalizable to women during their natural menopausal transition, only to some degree. Recruitment was based on similarities in demographic factors between the study population and the target regions. Yet, the target area was comprised of a limited number of women of race/ethnicities other than black and white. Consequently, in the MWHS, a total of 780 women were enrolled with a dominant representation of black (32%) and white (67%) women. Hence, the MWHS has identified specific factors that may be useful in informing the hot flash experience for these two racial/ethnic groups.

Additionally, many participants were going through their menopausal transition, a period that is characterized by irregular menstrual cycles. Therefore, biological samples were not collected on a specific day or phase of the menstrual cycle. To minimize biases that occur with the collection of biological samples at random time points, the participants were asked on a yearly basis to donate samples each week during four consecutive weeks. Hormone levels of these samples were averaged for each participant over these 4 weeks for each year of participation. Lastly, during the study duration, women who reported having at least one of the initial exclusion criteria (e.g., pregnancy, taking hormone replacement therapy) were discontinued from the study. Their data were not used from the time of reporting having one of these criteria and onward. This approach was taken because the MWHS was originally designed to identify risk factors (including hormone levels) related to natural occurring hot flashes. These participants were discontinued regardless if they reported having hot flashes or not, and only a relatively small number of participants were discontinued.

The MWHS also has several notable strengths. First, the MWHS included a relatively large sample size with a fairly high retention rate over the study years. It is also one of the few studies to have collected detailed data regarding the dynamics of past and present experience of hot flashes. Although hot flashes history was self-reported, this approach is similar to what was used in other population-based studies and is a valid indicator of hot flashes that is accepted by the National Institute of Health [11, 51, 52]. Further, during the MWHS, multiple biological samples, general measurements (e.g., weight, blood pressure), and other data from the participants were collected. This allowed the examination of specific changes over the study period (e.g., change in weight, lifestyle habits) and their association with menopausal hot flashes.

## Conclusion

The MWHS followed hundreds of women over several years during their menopausal transition by collecting multiple biological samples and measurements and self-reported questionnaire data. The study was designed and conducted by an experienced research team using well-established research tools. The MWHS results contribute a substantial amount of information relevant to women during their menopausal transition and clinicians who take care of these women. Some of the findings include the need to raise public awareness of changes in lifestyle habits that may alleviate menopausal hot flashes. The MWHS recently ended recruitment and data are still being analyzed. Therefore, more findings will be reported in the future.
